# Observability of Boolean multiplex control networks

**DOI:** 10.1038/srep46495

**Published:** 2017-04-28

**Authors:** Yuhu Wu, Jingxue Xu, Xi-Ming Sun, Wei Wang

**Affiliations:** 1 School of Control Science and Engineering, Dalian University of Technology, Dalian 116024, P.R. China; 2 Key Laboratory of Ocean Energy Utilization and Energy Conservation of Ministry of Education, Dalian University of Technology, 116024, Dalian, China

## Abstract

Boolean multiplex (multilevel) networks (BMNs) are currently receiving considerable attention as theoretical arguments for modeling of biological systems and system level analysis. Studying control-related problems in BMNs may not only provide new views into the intrinsic control in complex biological systems, but also enable us to develop a method for manipulating biological systems using exogenous inputs. In this article, the observability of the Boolean multiplex control networks (BMCNs) are studied. First, the dynamical model and structure of BMCNs with control inputs and outputs are constructed. By using of Semi-Tensor Product (STP) approach, the logical dynamics of BMCNs is converted into an equivalent algebraic representation. Then, the observability of the BMCNs with two different kinds of control inputs is investigated by giving necessary and sufficient conditions. Finally, examples are given to illustrate the efficiency of the obtained theoretical results.

Human Genome Project, which is an international scientific research project with the goal of determining the sequence of nucleotide base pairs^1^, inspired a new view of biology called the systems biology. Instead of investigating individual genes, proteins or cells, systems biology studies the behavior and relationships of all cells, proteins, DNAs and RNAs in the same biological system, called a cellular network^2^. The Boolean Networks (BNs) as a powerful tool in describing, analyzing, and simulating the cellular networks, has been most widely used^3^,^4^,^5^,^6^,^7^,^8^,^9^,^10^,^11^,^12^,^13^,^14^,^15^,^16^,^17^.

From decades ago, Kauffman put forward the theory which can describe the net of cell and gene using BNs^4^. And he made expatiation about the relationship between BNs and gene as well as life^5^,^6^. Because the construction and evolutionary process of cell and gene can be revealed very well by BNs, BNs turn into a hot topic concerned by biologists, physicists and neuroscientists. Huang, S. *et al*. talked about the Boolean modeling and analyzing of biological system^10^,^11^. Aldana, M. *et al*. studied the topological structure of BNs^7^. Akutsu, T. *et al*. and Albert, R. *et al*. considered the dynamic features of BNs^12^,^13^. More detailedly and recently, Lu, J. *et al*. analyzed the synchronization problem of master-slave probabilistic BNs^18^.

In recent years accompany with the development of biology, control of biological system becomes into a hot topic^19^,^20^,^21^,^22^,^23^,^24^,^25^,^26^,^27^,^28^,^29^. As to the research of genetic regulatory networks (GRNs), one of the major goals is to carry out the therapeutic intervention strategies for diseased targets^30^,^31^. Correspondingly, Boolean control networks (BCNs) as a theoretical branch of the above studies provide an efficient way to investigate the control of GRNs based on abstract models. So the interests to the BCNs are increasingly going up. The application of BCNs includes not only GRNs^32^, but also other various fields, such as man-machine dynamic game^33^ and internal combustion engines^34^,^35^. Recently, based on semi-tensor product (STP) proposed by Cheng, D. *et al*.^36^, many basic problems for BCNs have been investigated, for example, realization^23^, controllability^24^,^26^, optimal control^15^,^33^, etc.

Observability of a system is a structural property. It is also a fundamental concept in control theory and systematic science and, not surprisingly, it has found many applications in systems biology. As early as 1976, Cobelli *et al*. studied controllability, observability and structural identifiability of biological compartmental systems of any structure^37^. In evolutionary dynamics, observability is the key to study whether the genetic process itself can be recovered from measurements of phenotypic characteristics^38^. Observability analysis is a necessary preliminary step to the design of observers, that is, systems that provide an estimate of the complete internal state based on measurements of the inputs and outputs^39^. There have been many studies on the observability of BCNs in recent years. Cheng, D. *et al*. have investigated the controllability and observability of BCNs^24^. Li, F. *et al*. have studied the observability of time-delayed BCNs^25^. Laschov, D. *et al*. have considered the observability of BNs through a graph-theoretic approach^39^. Zhang, K. *et al*. have proposed a unified approach to determine all the four types of observability of BCNs in the literature^40^.

From the view of systems biology, the analysis in system-level of biological regulation needs to consider the interactions of genes on a holistic level, rather than the independent characteristics of isolated parts of an organism^41^. To understand the intricate variability of biological systems, where many hierarchical levels and interactions coexist, a new level of description is required. Thereupon, multiplex networks as an extension of complex networks were firstly proposed by Mucha in 2010^42^, which is composed of several layered networks interrelated with each other shown in Fig. 1. The previous description implicitly assumes that all biochemical signals are equivalent and then collapses information from different pathways. Actually, in cellular biochemical networks, many different signaling channels do work in parallel^43^. Not only in cellular biochemical, multiplex networks have been applied to the natural, social, and information sciences^42^. As an old concept, multi-layer social networks have been studied from 1975^44^. Transportation systems are natural candidates for a multi-layer network representation. In a recent paper, a two-layer structure has been created by merging the world wide port and airport networks^45^. In multiplex networks, each layer could have particular features and dynamical processes. Between layers, interconnections are represented by some special nodes on behalf of different roles participating in multiple layers of interactions. The final states of those common nodes at each time are determined by all involved layers, which is different from the traditional sense of coupling.

Recent years more and more researchers studied the BMNs. For example, Xu, M. *et al*. investigated the synchronizability of two-layer networks^46^. Cozzo talked about the stability of BMNs^43^. Luo *et al*. studied the controllability of BMCNs^27^. Zhong, J. *et al*. studied controllability problem for multi-level Boolean control networks^47^. But when it comes to the observability problem of BMCNs, to our best knowledge, there have been even no results, because there are many differences between BMNs and single-layer BNs. Even for the degenerated BMNs, their observability are different from the single-layer BNs’, for example the BCNs studied by Cheng, D. *et al*.^24^ and Li, F. *et al*.^25^. Because even when the number of layer is one, our system still has holistic states, which have logical relationship with the states in basic layers. From above discussions, we can know that a study of the observability of the BMNs is meaningful and challenging.

In this article, by following this stream of research, we first address and characterize observability of BCNs defined on multiple topological layers. Based on the model of Boolean multiplex networks presented by Coozo *et al*.^43^, we introduce the input controls and the outputs. The model of BMCNs are changed into algebraic representation using STP tools. We consider the observability of BMCNs, following the standard formulation of the observability problem in systems theory, namely, we assume that the BN structure is known and that the goal is to infer the initial condition based on an output sequence. To clearly show the results of our research, we gave observable and unobservable examples in the final part of our essay.

The rest of this article is organized as follows. In Section II, we introduce the dynamic structure of BMCNs. In Section III, some concepts and properties of the STP are introduced, and we change our network into discrete form using STP tool. In Section IV, necessary and sufficient conditions for the observability of the BMCNs are obtained. Examples are given to show the effectiveness of the obtained results in Section V. Finally, a brief summary is given in Section VI.

## Model of BMCNs

**Figure 1:**
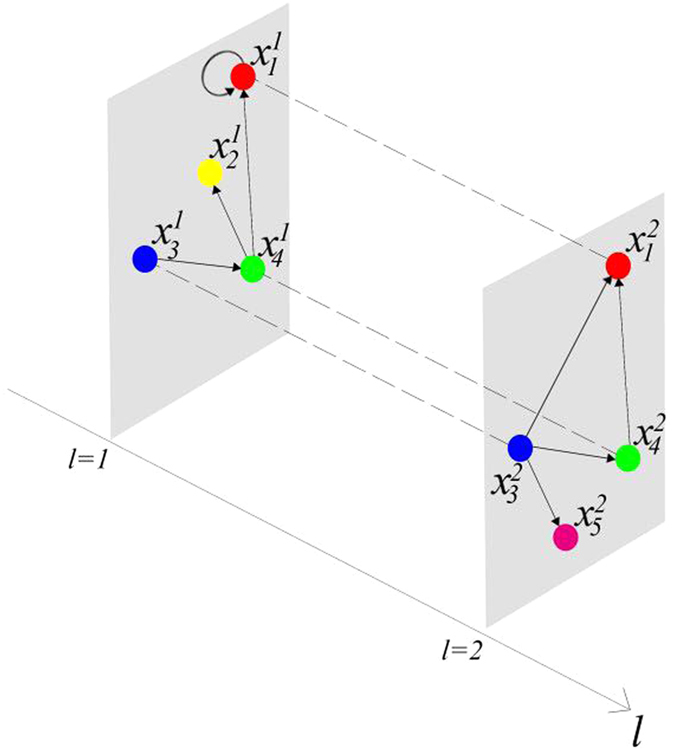
Schematic of multiple networks with two layers. Here *K* = 2 means that the system is a two layers network. *N* = 4 means that there are four nodes in each layer. And *n* = 5 means that there are five total different nodes in the system. 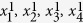 are the states in layer 1, and 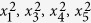 are the states in layer 2.

In this section, we introduce the model of BMCNs. For multiplex networks, different from the single-layer model, some nodes exist in multiple layers, the states which on different layers evolve independently of each other. The multiplex network we defined has *N* nodes per layer and *K* layers, and the number of total different nodes is *n (N* ≤ *n* ≤ *NK*). For example in Fig. 1, we have that *N* = 4, *K* = 2, *n* = 5. For statement ease, we define some related notions.

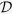
 is the set of {0, 1}.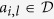, and *a*_*i,l*_ = 1 if node *i* in the *l* layer and 0 otherwise. The layers set of node *i* is 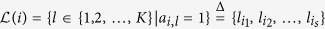 which refers the set of *l* which has *a*_*i,l*_ = 1.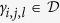, and *γ*_*i,j,l*_ = 1 if node *j* is the incoming neighbors of node *i* in the *l* layer. The incoming neighbors set of node *i* in the *l* layer is 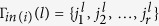 which refers the set of *j* which has *γ*_*i,j,l*_ = 1. And we set 
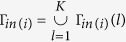
.

In Fig. 1, we have that the layers set of node 1 is 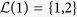, and *a*_1,1_ = 1 and *a*_1,2_ = 1, the layers set of node 2 is 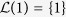, and *a*_2,1_ = 1 and *a*_2,2_ = 0. The incoming neighbors set of node 1 in layer 1 is Γ_*in*(1)_(1) = {1,4}, and 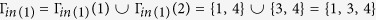.

In each layer, for the specific 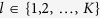, if *a*_*i,l*_ = 1, we assume 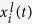 represents the state of node *i* on layer *l* at time *t*, then the update dynamics of state 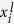 can be described as


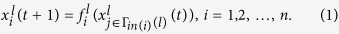


where 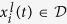, 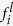 is the update rule of node *i* on layer *l*.

**Figure 2:**
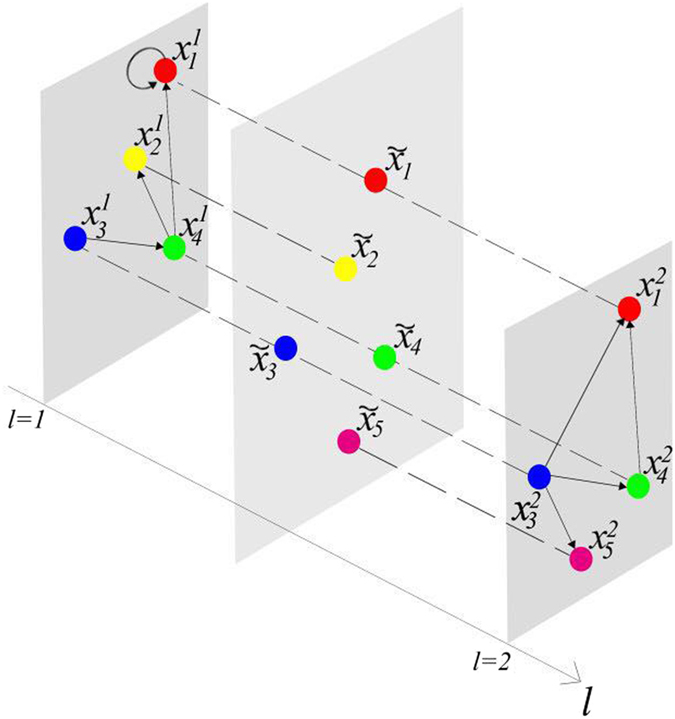
Schematic illustration of the relationship of node states in the fixed layers with the holistic states in BMNs. Where, 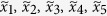 represent the holistic states of BMNs. For example 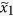 is the holistic state of 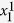 and 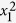. It is affected by 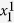 and 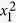 through canalizing function 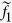. The second node is only existed in layer one. So holistic state 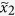 is only affected by 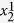.

Furthermore, assume 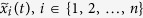 represents the holistic states of node *i* at time *t*, which means the global states of 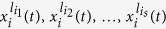, see Fig. 2. 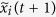 is influenced by 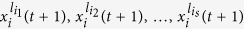, so we describe it as


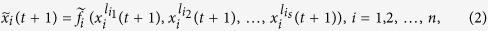


where.. is the canalizing function.

**Figure 3:**
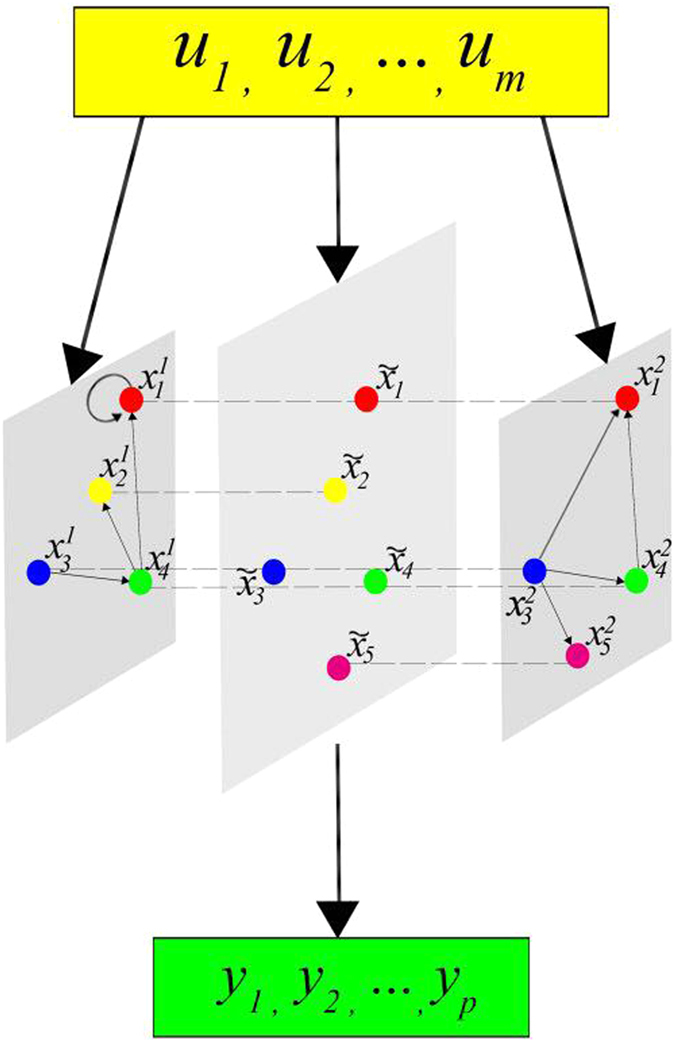
Schematic illustration of BNs with control and output. The inputs *m* dimension control 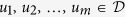 have been introduced. 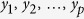 denote outputs. From the figure, we see that inputs 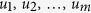 affect the node states in each layers as well as the abstract holistic states. And outputs 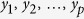 are affected by holistic states 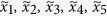.

When considering control-related problems for BMNs, based on above system structure, we introduce the *m*-dimensional control 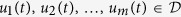 as the inputs of the system, correspondingly, then we have the outputs 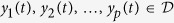, then the BMCNs can be described as


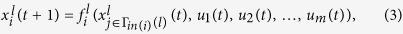


and


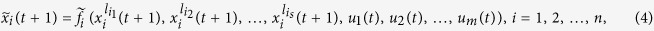


where 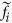 is the canalizing function of node *i* with the controls 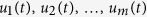, see Fig. 3.

In finally, the output dynamics of the BMCNs are given by the following equation


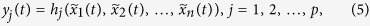


where *h*_*j*_ is the output function.

**Remark 1.** The BMCNs are not the simple superposition of *K* single-layer BCNs. Because we have the holistic states which are affected by corresponding states in each layers. Between the holistic states with the states in basic layers, we have the canalizing functions, which determine the value of the holistic states. Even when the number of layer is one, our system still has holistic states, which have logical relationship with the states in basic layers through the canalizing functions. So it is still different from the one layer BNs.

## Algebraic representation of BMCNs

In this section, we introduce some concepts and properties, changing our BMCNs into algebraic representation.

### Concepts and properties of the semi-tensor product

In this subsection, some concepts and properties of the STP will be briefly introduced^36^.

**Definition 1.**^36^

- Let *X* be a row vector of dimension np, and *Y* = [*y*_1_, *y*_2_,…, *y*_*p*_]^*T*^ be a column vector of p dimension. Then we split *X* into p equal-size blocks as X^1^, *X*^2^, …, *X*^*p*^, which are 1 × n rows. Define the STP, denoted by 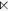, as
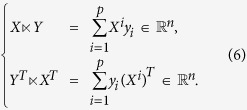
- Let 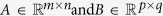. If either n is a factor of p, say nt = p and denote it as 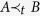, or p is a factor of n, say n = pt and denote it as 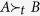, then we define the STP of A and B, denoted by 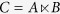, as the following: C consists of m × q blocks as C = (*C*^*ij*^) and each block is
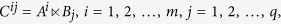

*where A*^*i*^ is the i–th row of *A* and *B*_*j*_ is the j–th column of *B*.

And here we give some fundamental properties of the STP in the following^36^:

**Lemma 1.**^36^ Assume 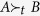, then (where ⊗ is the Kronecker product, *I*_*t*_ is the identity matrix).


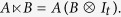


Assume 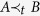, then


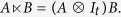


**Lemma 2.**^36^ Assume A ∈ *M*_*m*×*n*_ is given.

- *Let Z* ∈ *R*^*t*^ be a row vector. Then
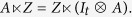
- *Let Z* ∈ *R*^*t*^ be a column vector. Then
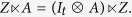

It is easy to find out that STP of matrix can be seen as a generalization of conventional matrix product. All the fundamental properties of conventional matrix product, such as distributive rule, associative rule, remain true. So we can omit the symbol 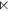.

Here we defined some notions for statement ease.

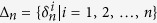
, where 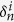 denotes the *i*–*th* column of the identify matrix *I*_*n*_.
- Assume a matrix 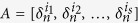, where 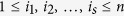 are positive integer constants. We can briefly denoted it as 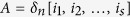.
- A matrix 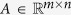 is called a logical matrix if the columns of *A*, denoted by *Col (A*), satisfy 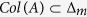. And the set of *m* × *n* logic matrices is denoted by 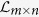.

Then we define a swap matrix 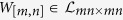, which is constructed in the following way: label its columns by (11, 12, …, 1*n*, …, *m*1, *m*2, …, *mn*) and its rows by (11, 21, …, *m*1, …, 1*n*, 2*n*, …, *mn*). And its element in the position ((*I, J*), (*i, j*)) is assigned as


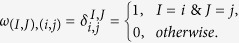


And we denote *W*_[*n*]_ = *W*_[*n, n*]_ when *m* = *n*.

**Lemma 3.**^36^ Let 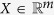 and 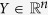 be two columns. Then


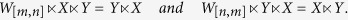


For the logical function with *n* arguments 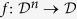, we can convert it into an algebraic function using the STP of matrices. A logical domain, denoted by 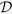, is defined as 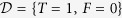. We identify each element in 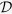 with a vector as 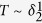, 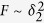 and 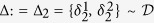. Based on this, we have

**Lemma 4.**^36^ Any logical function f(*x*_1_, *x*_2_, …, *x*_*n*_) with logical arguments x_1_, *x*_2_, …, *x*_*n*_ ∈ Δ, can be expressed in a multi-linear form as


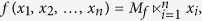


where 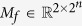 is unique, which is called the structure matrix of logical function f.

And here we give another lemma:

**Lemma 5.**^36^ *Assume* 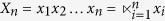, *then*


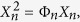


where


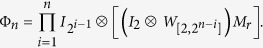


Here *M*_*r*_ = *δ*_4_[1, 4], which is power-reducing matrix and it can be verified that *P*^2^ = *M*_*r*_*P*, ∀*P* ∈ Δ.

Based on the above properties of STP, we put forward an obvious proposition.

**Proposition 1.** *For each* 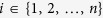, *if* 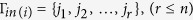, *we can find a matrix R*_*i*_ *such that*


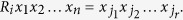


### Algebraic structure of the BMCNs

In this subsection, we change our BMCNs into discrete version using STP tool. To express it more clearly, here we give some description of variables.

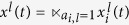
 means the state of layer *l*.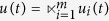 means the control.
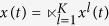
 means the state of all layers.

In the following step we will change the given BMCNs (3)-(4) into algebraic representation, as we will find out the algebraic relation between *x(t* + 1) and *x(t*) as well as the algebraic relation between 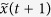 and *x(t*).

At the first place, we will find out the algebraic relation between *x(t* + 1) and *x(t*). Using lemma 4 and proposition 1, for each logical rule 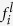, we can find its structure matrix 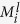, so we obtain that


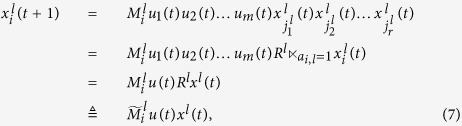


where 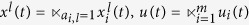, and 
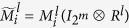
. Hence


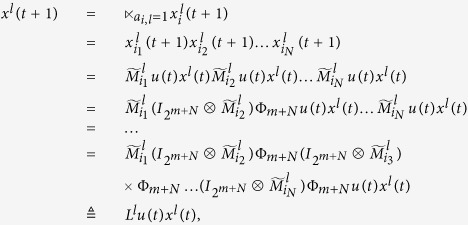


with 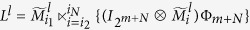. And we have defined that 
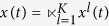
, so we obtain that


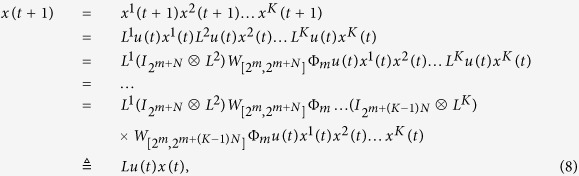


where 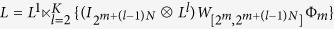.

Subsequently, we will find out the algebraic relation between 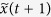 and *x(t*). Using the similar steps above, the algebraic representation of (4) can be obtained as


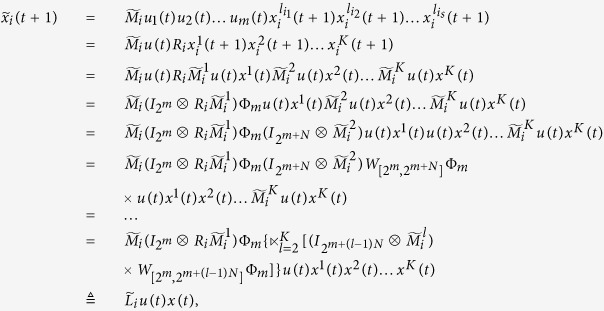


where 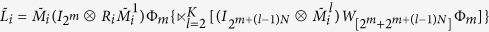 is the structure matrix of logical function 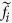. And we have that 
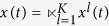
. So we obtain that


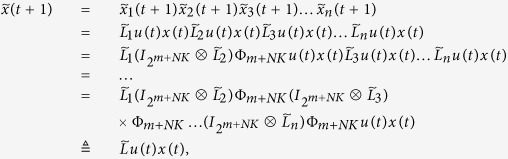


where 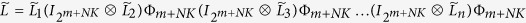.

Means that


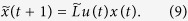


Similarly, by letting 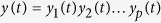, we obtain the algebraic expression of the output dynamics (5) as follows:


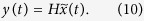


where 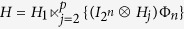, here *H*_*j*_s are the structure matrixes of *h*_*j*_, *j* = 1, 2, …, *p*.

Here we give an example to illustrate this process.

**Example 1.** Consider following two-layer BMCN, *with N* = 2, *K* = 2, *n* = 4 *and m* = 4


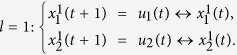



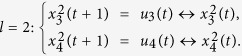


and we have that


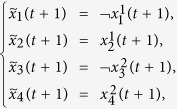


where 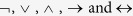 represent the logical functions of negation, disjunction, conjunction, implication, and equivalence, respectively. Based on Lemma 4, we obtain the corresponding structure matrices of those logical operators, as given in Table 1.

Define 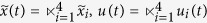. Then we calculate the control-depending network transition matrix of system.


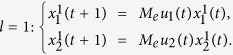



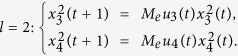


and we have that


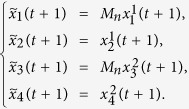


Then


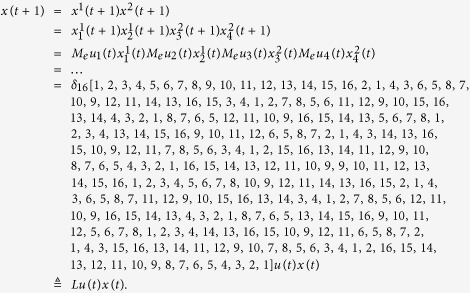



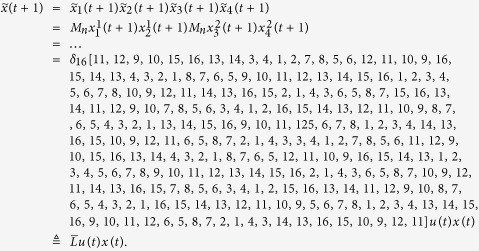


Here we have found out the algebraic relation between x(*t* + *1*) and *x(t*) as well as the algebraic relation between 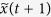 *and x(t*). Furthermore, we assume that


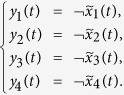


Then, according to properties of STP, we obtain the matrix expression of output, as follows


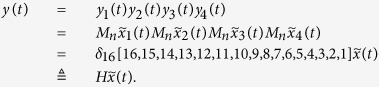


## Observability of BMCNs

In this section, we will analyze and characterize the observability of the BMCNs, with two different types of controls. We first provide some definitions as follows.

Consider the BMCN (3)-(4) with output dynamics (5). For any initial state 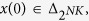 and control input sequence 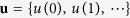, the holistic trajectory at time *t* is denoted by 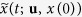. Output trajectory at time *t* denote by *y*(; **u**, *x*(0)).

**Definition 2.** The BMCN (3)-(5) is observable if there exists a finite control sequence 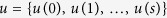, with s > 0, such that for any 
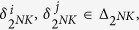
 with i ≠ *j*, we have 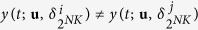 for some 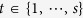.

In other words, there exists a control input sequence for which the initial state can be uniquely determined from the knowledge of the output sequence.

**Remark 2.** Our definition is motivated by the definition of observability for BCNs proposed in Laschov, *et al*.^39^, which is different from the one proposed by Cheng, D. *et al*.^24^. In Cheng, D. *et al*.^24^, a BCN is said to be observable if the initial state can be uniquely determined from the knowledge of the control inputs (which may depend on the initial state) and the outputs.

We consider two kinds of controls. The first is that the controls are determined by certain logical rules, which called the input networks.


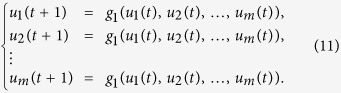


where 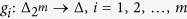, are logical function.

According Lemma 4, we know that the input network (11) can be expressed as


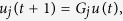


where 
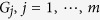
 are the structure matrix of logical function *g*_*i*_, respectively. Then,


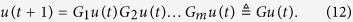


with 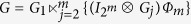.

**Theorem 1.** Consider (3)-(5) (or equivalently (8)-(10)) with input network control (11). The system is observable if and only if there exists finite time s, *s* > 0, such that 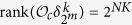, for some 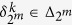, where


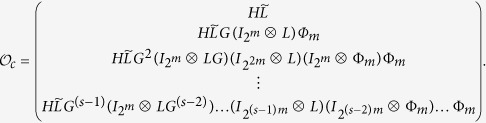


*Proof*. By considering the input network, put together (8)-(9) with (12), we can obtain the system


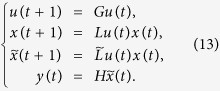


A straightforward computation shows, we calculate the output 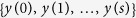 as follows


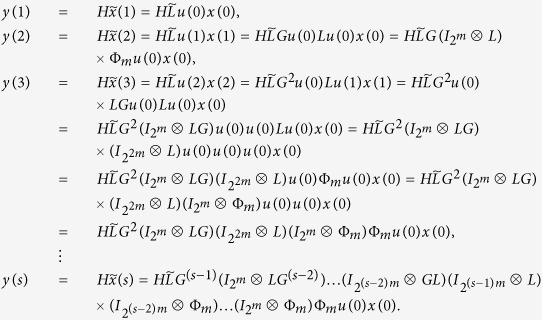


Hence, in the matrix form, we obtain


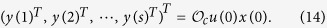


From the solution structure of the system of linear algebraic equations, we know that for some initial control input 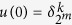, the system of linear equations (14) with 2^*NK*^-dimension unknown vector *x*(0) has a unique solution if and only if the system matrix 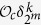 has rank 2^*NK*^. That is, for some initial control input 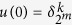, the initial state *x*(0) is uniquely determined by the knowledge of the output sequence 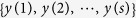 if and only if


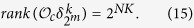


This completes the proof.

**Remark 3.** From the proof of above theorem, we obtain that for some 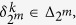 if the matrix 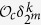 has full column rank, means that 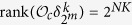, then the initial state x(0) can be reconstructed by the left inverse of 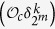 operation on output sequence 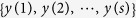,


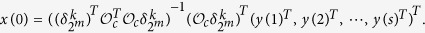


In the following, we consider the case when the controls are free Boolean sequence. Precisely, *m* controls are described as 
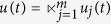
 and freely designed.

**Theorem 2.** Consider (3)-(4) and (5) (or equivalently (8), (9) and (10)), with a free Boolean sequence control. The system is observable if and only if there exists a finite control sequence 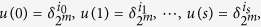 with 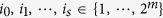 such that 
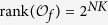
, where


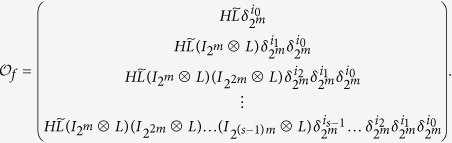


*Proof*. If the controls come from a free Boolean sequence, the system is that


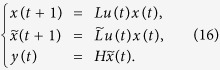


If free control inputs 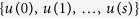 are given, then a straightforward computation shows the following:


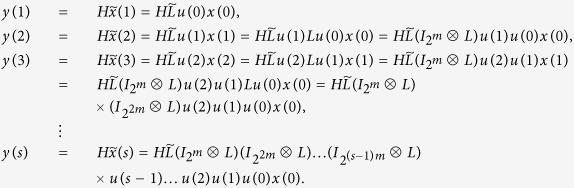


Hence, in the matrix form, we obtain


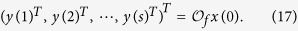


As a similar analysis discussed in the proof of Theorem 1, we know that for a given free control inputs 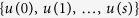, the system of linear equations (17) with 2^*NK*^-dimension unknown vector *x*(0) has a unique solution if and only if the system matrix 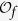 has rank 2^*NK*^. That is, for a given free control inputs 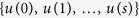, the initial state *x*(0) is uniquely determined by the knowledge of the output sequence 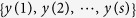 if and only if 
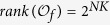
. Furthermore, as mentioned in Remark 3, the initial state *x*(0) can exactly calculated as 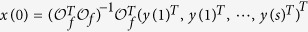 This completes the proof.

**Remark 4.** The observability in our paper is the observability of 
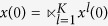
 which is the all initial states of all layers in the initial time. Boolean control network (3)-(4) is observable if for the initial state 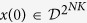, there exists finite time 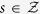, such that the initial state can be uniquely determined from the knowledge of the controls 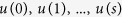 and the outputs 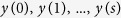. Based on the initial state 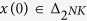, we can easily obtain the holistic states 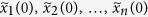 through the canalizing function 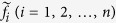. So the holistic states 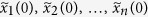 are also observable.

## Examples

In this section, we will give some examples to illustrate our results. Example 2 is a observable case and Example 3 is an unobservable case.

**Example 2.** (Continue to Example 1) Consider the two-layer BMCN given in Example 1. Assume that the control inputs are determined by the following input network


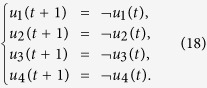


Then we obtain that


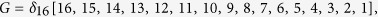


If we take 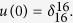 By calculation, we have


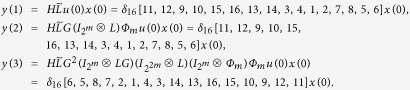


Then we have that


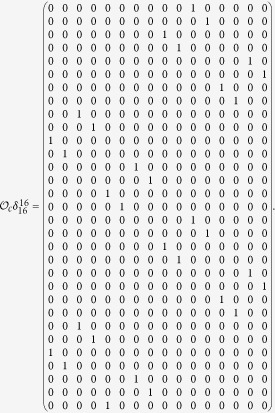


And we can obtain that 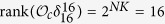. Then from Theorem 1, we know that the system is observable under the input network (18).

**Example 3.** Consider following two-layer BMCN, with *N* = 2, *K* = 2, *n* = 3 and *m* = 1


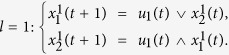



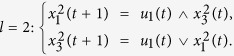


and we have that


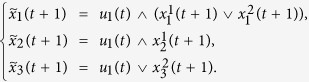


Then we calculate the control-depending network transition matrix of system.


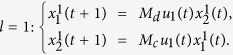



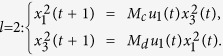


and we have that


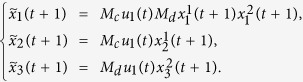


Then


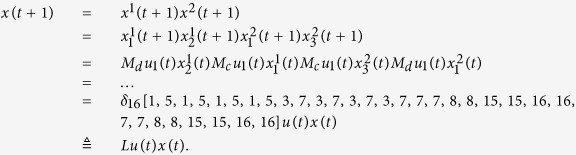



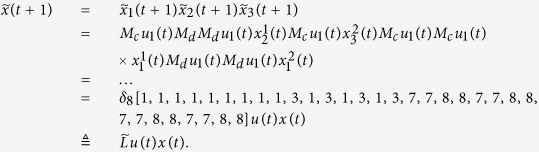


Furthermore, we assume that


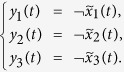


Then, according to properties of STP, we obtain the matrix expression of output, as follows


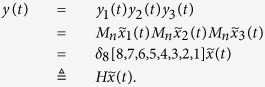


Now, we analyze the observability of this system, based on Theorem 2. We can calculate that while 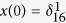,


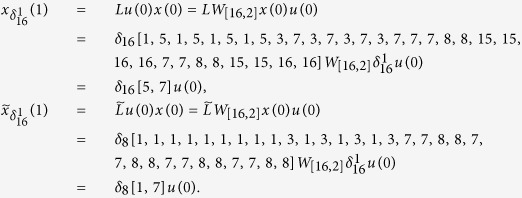


And while 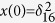, we have that


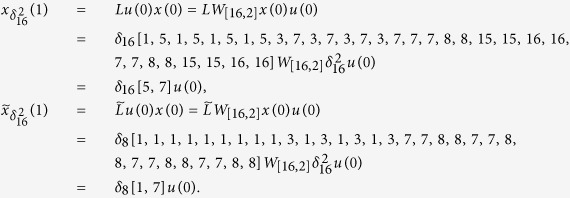


Then, by induction, we easy obtain that, for any s > 0, and free control input 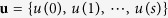, 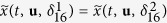, and furthermore, 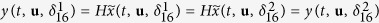. That implies, for any s > 0, and free control input 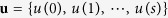



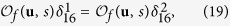


So the linear homogeneous equation


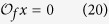


has the non-zero solution. Then we obtain that for arbitrary s > 0, we still have that 
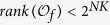
, by Theorem 2, the system is unobservable.

## Conclusions

In this paper, input controls were introduced into BMNs. By means of STP approach, the above logical dynamics has been converted into an algebraic form and the observability of dynamics is discussed. Firstly, we gave the theorem about the observability of whole dynamic system. Subsequently, the observability of each node in the special layer has been proved. Finally, we put forward some examples to illustrate our results.

## Additional Information

**How to cite this article:** Wu, Y. *et al*. Observability of Boolean multiplex control networks. *Sci. Rep.* **7**, 46495; doi: 10.1038/srep46495 (2017).

**Publisher's note:** Springer Nature remains neutral with regard to jurisdictional claims in published maps and institutional affiliations.

## Figures and Tables

**Table 1:** Structure matrices of some basic logical functions.

*f(x*_1_, *x*_2_)	¬*x*_1_	*x*_1_ ∨ *x*_2_	*x*_1 _∧_ _*x*_2_	*x*_1_ → *x*_2_	*x*_1_ ↔ *x*_2_
*M*_*f*_	*M*_*n*_ = *δ*_2_[2, 1]	*M*_*d*_ = *δ*_2_[1, 1, 1, 2]	*M*_*c*_ = *δ*_2_[1, 2, 2, 2]	*M*_*i*_ = *δ*_2_[1, 2, 1, 1]	*M*_*e*_ = *δ*_2_[1, 2, 2, 1]

## References

[b1] Hood L. & Rowen L. The human genome project: big science transforms biology and medicine. Genome Medicine 5, 1–8 (2012). 10.1186/gm483 PMC4066586 24040834

[b2] Kitano H. Systems biology: A brief overview. Science 295, 1662–1664 (2002). 11872829 10.1126/science.1069492

[b3] Zhong J., Lu J., Liu Y. & Cao J. Synchronization in an array of output-coupled boolean networks with time delay. IEEE Transactions on Neural Networks & Learning Systems 25, 2288–2294 (2014). 25420249 10.1109/TNNLS.2014.2305722

[b4] Kauffman S. A. Metabolic stability and epigenesis in randomly constructed genetic nets. Journal of Theoretical Biology 22, 437–467 (1969). 5803332 10.1016/0022-5193(69)90015-0

[b5] Kauffman S. A. & (OUP), O. U. P. Origins of order: self-organization and selection in evolution. Journal of Evolutionary Biology 13, 133–144 (1993).

[b6] Kauffman S. A. At home in the universe. Mathematical Social Sciences 33, 94–95 (1995).

[b7] Aldana M. Boolean dynamics of networks with scale-free topology. Physica D Nonlinear Phenomena 185, 45–66 (2003).

[b8] Heidel J., Maloney J., Farrow C. & Rogers J. A. Finding cycles in synchronous boolean networks with applications to biochemical systems. International Journal of Bifurcation & Chaos 13, 535–552 (2011).

[b9] Farrow C., Heidel J., Maloney J. & Rogers J. Scalar equations for synchronous boolean networks with biological applications. IEEE Transactions on Neural Networks 15, 348–354 (2004). 15384528 10.1109/TNN.2004.824262

[b10] Huang S. & Ingber D. E. Shape-dependent control of cell growth, differentiation, and apoptosis: Switching between attractors in cell regulatory networks. Experimental Cell Research 261, 91–103 (2000). 11082279 10.1006/excr.2000.5044

[b11] Huang S. Regulation of cellular states in mammalian cells from a genomewide view. In Gene Regulations and Metabolism - Postgenomic Computational Approaches 181–220 (2002).

[b12] Akutsu T., Miyano S. & Kuhara S. Inferring qualitative relations in genetic networks and metabolic pathways. Bioinformatics 16, 727–734 (2000). 11099258 10.1093/bioinformatics/16.8.727

[b13] Albert R. & Barabasi A. L. Dynamics of complex systems: scaling laws for the period of boolean networks. Physical Review Letters 84, 5660–5663 (2000). 10991019 10.1103/PhysRevLett.84.5660

[b14] Meng M. & Feng J. E. Synchronization of interconnected multi-valued logical networks. In Chinese Control Conference 1659–1669 (2013).

[b15] Wu Y. & Shen T. An algebraic expression of finite horizon optimal control algorithm for stochastic logical dynamical systems. Systems & Control Letters 82, 108–114 (2015).

[b16] Villegas P., Ruiz-Franco J., Hidalgo J. & Muñoz M. A. Intrinsic noise and deviations from criticality in boolean gene-regulatory networks. Scientific Reports 6, 34743 (2016). 27713479 10.1038/srep34743 PMC5054426

[b17] Chen H., Wang G., Simha R., Du C. & Chen Z. Boolean models of biological processes explain cascade-like behavior. Scientific Reports 7, 20067 (2016). 26821940 10.1038/srep20067 PMC4731822

[b18] Lu J., Zhong J., Li L., Ho D. W. & Cao J. Synchronization analysis of master-slave probabilistic boolean networks. Scientific reports 5, 13437 (2015). 26315380 10.1038/srep13437 PMC4551960

[b19] Chen H. & Sun J. A new approach for global controllability of higher order boolean control network. Neural Networks the Official Journal of the International Neural Network Society 39, 12–17 (2013). 23298550 10.1016/j.neunet.2012.12.004

[b20] Li F. & Sun J. Controllability of higher order boolean control networks. Applied Mathematics & Computation 219, 158–169 (2012).

[b21] Lu J., Zhong J., Ho D. W. C., Tang Y. & Cao J. On controllability of delayed boolean control networks. SIAM Journal on Control & Optimization 54, 475–494 (2016).

[b22] Lu J., Zhong J., Huang C. & Cao J. On pinning controllability of boolean control networks. IEEE Transactions on Automatic Control 61, 1658–1663 (2016).

[b23] Cheng D., Li Z. & Qi H. Realization of boolean control networks. Automatica (Journal of IFAC) 46, 62–69 (2010).

[b24] Cheng D., Qi H. & Li Z. Controllability and observability of boolean control networks. Automatica 45, 1659–1667 (2009).

[b25] Li F., Sun J. & Wu Q. D. Observability of boolean control networks with state time delays. IEEE Transactions on Neural Networks 22, 948–954 (2011). 21518658 10.1109/TNN.2011.2126594

[b26] Zhang L. & Zhang K. Controllability and observability of boolean control networks with time-variant delays in states. IEEE Transactions on Neural Networks & Learning Systems 24, 1478–1484 (2013). 24808585 10.1109/TNNLS.2013.2246187

[b27] Luo C., Wang X. & Liu H. Controllability of time-delayed boolean multiplex control networks under asynchronous stochastic update. Scientific Reports 4, 7522 (2014). 25516009 10.1038/srep07522 PMC4268650

[b28] Zhong J., Lu J., Huang T. & Ho D. W. C. Controllability and synchronization analysis of identical-hierarchy mixed-valued logical control networks. IEEE Transactions on Cybernetics, doi: 10.1109/TCYB.2016.2560240 (2016). 27323388

[b29] Ideker T., Galitski T. & Hood L. A new approach to decoding life: systems biology. Annual Review of Genomics & Human Genetics 2, 343–372 (2003). 10.1146/annurev.genom.2.1.343 11701654

[b30] Kadelka C., Murrugarra D. & Laubenbacher R. Stabilizing gene regulatory networks through feedforward loops. Chaos: An Interdisciplinary Journal of Nonlinear Science 23, 025107 (2013). 10.1063/1.4808248 23822505

[b31] Chaves M., Sontag E. D. & Albert R. Methods of robustness analysis for boolean models of gene control networks. Systems Biology 153, 154–167 (2006). 16986617 10.1049/ip-syb:20050079

[b32] Faryabi B., Datta A. & Dougherty E. R. On approximate stochastic control in genetic regulatory networks. IET Systems Biology 1, 361–368 (2007). 18203582 10.1049/iet-syb:20070015

[b33] Cheng D., Zhao Y. & Xu T. Receding horizon based feedback optimization for mix-valued logical networks. IEEE Transactions on Automatic Control 60, 3362–3366 (2015).

[b34] Wu Y., Kumar M. & Shen T. A stochastic logical system approach to model and optimal control of cyclic variation of residual gas fraction in combustion engines. Applied Thermal Engineering 93, 251–259 (2016).

[b35] Wu Y. & Shen T. Policy iteration approach to control residual gas fraction in ic engines under the framework of stochastic logical dynamics. IEEE Transactions on Control Systems Technology 25, 1100–1107 (2017).

[b36] Cheng D., Qi H. & Zhao Y. An Introduction to Semi-Tensor Product of Matrices and Its Applications (World Scientific Publishing Co. Pte. Ltd., 2012).

[b37] Cobelli C. & Romanin-Jacur G. Controllability, observability and structural identifiability of multi input and multi output biological compartmental systems. IEEE Transactions on Biomedical Engineering 23, 93–100 (1976). 1248848 10.1109/tbme.1976.324568

[b38] Lopez I., Gamez M. & Carreno R. Observability in dynamic evolutionary models. Biosystems 73, 99–109 (2004). 15013222 10.1016/j.biosystems.2003.10.003

[b39] Laschov D., Margaliot M. & Even G. Observability of boolean networks: A graph-theoretic approach. Automatica 49, 2351–2362 (2013).

[b40] Zhang K. & Zhang L. Observability of boolean control networks: A unified approach based on finite automata. IEEE Transactions on Automatic Control 61, 2733–2738 (2016).

[b41] Kitano H. Systems biology: a brief overview. Science 295, 1662–1664 (2002). 11872829 10.1126/science.1069492

[b42] Mucha P. J. & Onnela J. P. Community structure in time-dependent, multiscale, and multiplex networks. Science 328, 876–878 (2010). 20466926 10.1126/science.1184819

[b43] Cozzo E., Arenas A. & Moreno Y. Stability of boolean multilevel networks. Physical Review E Statistical Nonlinear & Soft Matter Physics 86, 2569–2575 (2012). 10.1103/PhysRevE.86.036115 23030988

[b44] Goffman E. Frame Analysis. An Essay on the Organization of Experience (PENGUIN, 1975).

[b45] Parshani R., Rozenblat C., Ietri D., Ducruet C. & Havlin S. Inter-similarity between coupled networks. EPL (Europhysics Letters) 92, 68002 (2010).

[b46] Xu M., Zhou J., Lu J.-a. & Wu X. Synchronizability of two-layer networks. The European Physical Journal B 88, 1–6 (2015).

[b47] Zhong J., Ho D. W. C., Lu J. & Xu W. Controllability for a special case of multi-level boolean control networks. In 2016 IEEE International Conference on Industrial Technology (ICIT) 1378–1383 (2016).

